# Supplementary figures and images for: Functionalized calcium carbonate microparticles in ethyl cellulose films: A vehicle for sustained amoxicillin release for medical applications
Source: PLoS One. 2026 Apr 2;21(4):e0320280. doi: 10.1371/journal.pone.0320280 (PMC13046161; doi:10.1371/journal.pone.0320280)

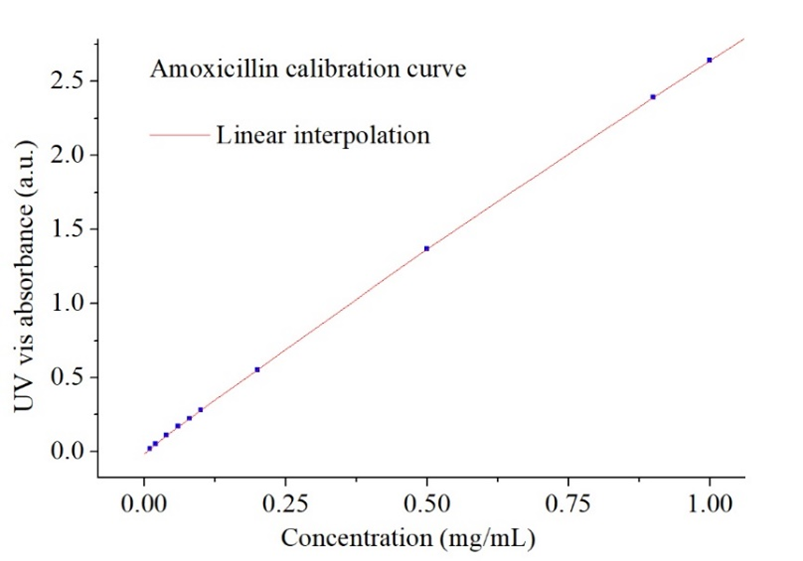

Supplement: S1 Fig — (TIF) [file pone.0320280.s001.tif]

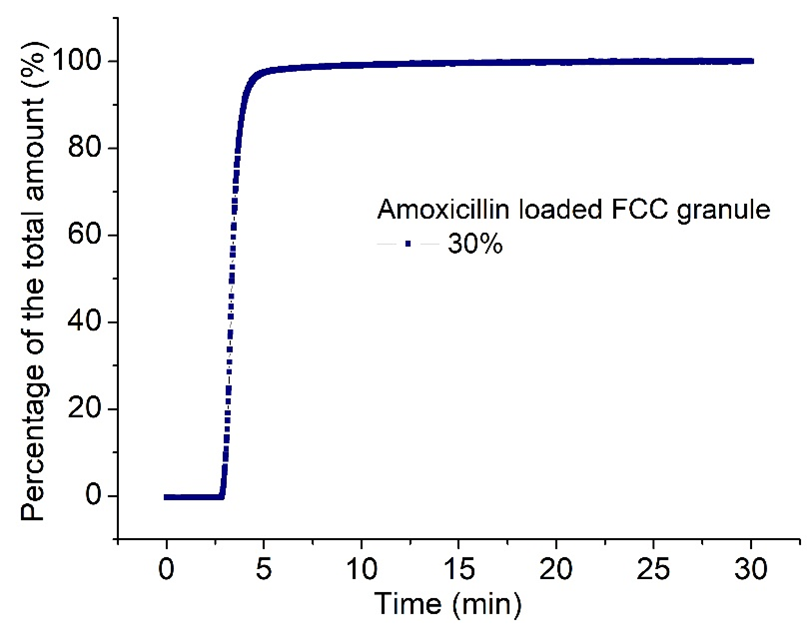

Supplement: S2 Fig — (TIF) [file pone.0320280.s002.tif]
