# Supplementary material for: Functionalized calcium carbonate microparticles in ethyl cellulose films: A vehicle for sustained amoxicillin release for medical applications
Source: PLoS One. 2026 Apr 2;21(4):e0320280. doi: 10.1371/journal.pone.0320280 (PMC13046161; doi:10.1371/journal.pone.0320280)
Supplement: S1 Table — (DOCX) [file pone.0320280.s003.docx]

| Aimed loading level (w/w%) | Measured loading (w/w%) | After release (w/w%) |
| --- | --- | --- |
| 15 | 12,8 | 0 |
| 30 | 21,8 | 0 |
