# Supplementary material for: Functionalized calcium carbonate microparticles in ethyl cellulose films: A vehicle for sustained amoxicillin release for medical applications
Source: PLoS One. 2026 Apr 2;21(4):e0320280. doi: 10.1371/journal.pone.0320280 (PMC13046161; doi:10.1371/journal.pone.0320280)
Supplement: S2 Table — (DOCX) [file pone.0320280.s006.docx]

| **Experiment** | **Sample Type** | **Sink vs. Non-sink** | **Release Medium Volume** | **Temp.** | **Agitation / Flow Conditions** | **Exposed Surface Area** |
| --- | --- | --- | --- | --- | --- | --- |
| **Particle release (Figs 3–4)** | FCC fines or FCC granules loaded with amoxicillin | Sink conditions | 75 mL Milli-Q water | 22 °C | Mechanical stirring at 100 rpm; continuous circulation through UV-Vis flow cell using peristaltic pump | Not applicable (dispersed particles) |
| **Film release – loaded FCC–EC film (Figs 8–9)** | EC film containing 10 w/w% Omyapharm (loaded with 30 w/w% amoxicillin) | Non-sink conditions | 5 mL Milli-Q water | 22 °C | No agitation (static); samples manually withdrawn and returned | 5.4 cm²/mL (coiled film with perforation geometry) |
| **Film release – physical mixture reference (Figs 8–9)** | EC film containing physical mixture of amoxicillin + Omyapharm | Non-sink conditions | 5 mL Milli-Q water | 22 °C | No agitation (static); samples manually withdrawn and returned | 5.4 cm²/mL (coiled film with perforation geometry) |
