# Supplementary material for: Functionalized calcium carbonate microparticles in ethyl cellulose films: A vehicle for sustained amoxicillin release for medical applications
Source: PLoS One. 2026 Apr 2;21(4):e0320280. doi: 10.1371/journal.pone.0320280 (PMC13046161; doi:10.1371/journal.pone.0320280)
Supplement: S1 Text — (DOCX) [file pone.0320280.s007.docx]

Classical release models such as Higuchi and Korsmeyer–Peppas assume sink conditions, constant diffusivity, and a single dominant release mechanism. The long-term film experiments in this study were intentionally conducted under non-sink conditions to mimic diffusion-limited environments surrounding indwelling medical devices. As a result, the increasing amoxicillin concentration in the medium alters the concentration gradient over time, violating fundamental model assumptions. Furthermore, the film exhibits a biphasic profile—an initial burst followed by a slow multi-month release—arising from distinct mechanisms that cannot be described by a single-regime kinetic model. For these reasons, applying Higuchi or Korsmeyer–Peppas fits would not yield meaningful or physically reliable parameters, and such analyses are therefore not included.
