# Supplementary material for: Functionalized calcium carbonate microparticles in ethyl cellulose films: A vehicle for sustained amoxicillin release for medical applications
Source: PLoS One. 2026 Apr 2;21(4):e0320280. doi: 10.1371/journal.pone.0320280 (PMC13046161; doi:10.1371/journal.pone.0320280)
Supplement: Attachment — Submitted filename: review letter- PONE-D-25-08617-R1 (1)_anon.pdf [file pone.0320280.s010.pdf]

General Assessment:

This study investigates FCC microparticles integrated in ethyl cellulose films for sustained amoxicillin delivery—an innovative and clinically relevant concept targeting catheter-related infections. The combined use of SEM, UV-vis, synchrotron XRD, and TGA strengthens the material analysis. Nonetheless, the manuscript currently falls short in methodological completeness, clarity, and novelty justification.

Major Issues (Essential for Revision):

1. **Material transitions and clarity**
   - The manuscript frequently switches between FCC fines, granules, and Omyapharm (e.g., in Chapters 2 and 3). A comparative table listing surface area (BET), morphology, rationale, and selection criteria for each form would greatly improve clarity.
2. **Film characterization for application**
   - Films designed for indwelling use require mechanical (tensile strength), swelling, and mucoadhesion data. At minimum, quantitative swelling or tensile information should be included, or limitations acknowledged.
3. **Drug release quantification**
   - Relying on TGA for drug release estimation is unconventional. While UV-vis release curves are present, detailed release kinetics (e.g., cumulative release, release rate ± SD, model fitting) must be shown. Clarify that TGA data are supplementary and residual only.
4. **Novelty and comparative context**
   - Similar systems (e.g., Mg-HA, Eudragit, MCM-41) have been used for amoxicillin delivery. The manuscript should explicitly highlight what is novel—such as use of FCC in EC films—and compare performance metrics (release duration, loading capacity) to existing systems.
5. **Terminology consistency**
   - Use “sustained” exclusively throughout. Currently, the manuscript alternates between “sustained” and “extended.” Ensure consistent usage.

Minor Comments:

- Correct remaining typographical and formatting issues (e.g., “Formatted: Font: 16 pt”).
- Expand figure legends (e.g., labeling apparatus components in Figure 2).
- Define all solvents and abbreviations in the Methods section first mention.
- Clarify that ethyl cellulose and FCC are biocompatible and sterile, as relevant to medical application.

Recommendation: Major Revision
