# Supplementary material for: Functionalized calcium carbonate microparticles in ethyl cellulose films: A vehicle for sustained amoxicillin release for medical applications
Source: PLoS One. 2026 Apr 2;21(4):e0320280. doi: 10.1371/journal.pone.0320280 (PMC13046161; doi:10.1371/journal.pone.0320280)
Supplement: Attachment — Submitted filename: review letter- revised version.pdf [file pone.0320280.s014.pdf]

Dear Chief Editor,

I have reviewed the revised manuscript entitled
“Functionalized Calcium Carbonate Microparticles in Ethyl Cellulose Films: A Vehicle for Sustained Amoxicillin Release for Medical Applications” (Manuscript ID: PONE-D-25-08617R3).
The authors have responded to the major concerns raised in the previous review, and the manuscript has improved in clarity and scope. The study remains a technically sound proof-of-concept for sustained amoxicillin release from FCC–ethyl cellulose composite films; however, several limitations and minor issues should still be carefully addressed before final acceptance.

General Assessment

The revised version more clearly defines the FCC materials used, improves terminology consistency, and better contextualizes the novelty of the system relative to prior amoxicillin delivery platforms. The scope of the work is now more appropriately framed, with explicit acknowledgment that the study does not yet represent a fully validated medical device.

Remaining Issues and Minor Comments

1. **Statistical and quantitative language**
   Long-term release experiments are still based on single representative samples. While this limitation is acknowledged, language such as *“average release rate”* should be softened to avoid over-interpretation. Terms such as *“apparent”* or *“estimated”* release rate would be more appropriate in this context.
2. **Figure clarity**
   Figures 8 and 9 would benefit from clearer, more explicit labeling of *“loaded”* versus *“physical mixture”* directly on the plots to improve readability without requiring extensive reference to the captions or text.
3. **Formatting and presentation**
   A small number of formatting artifacts and spacing inconsistencies remain in the revised manuscript and should be corrected during final editing.
4. **MIC and biological relevance**
   The discussion linking sustained release behavior to MIC values is informative; however, it should remain cautiously framed as indicative only. Care should be taken to avoid implying predictive relevance for in vivo efficacy at this stage.

Recommendation

Minor Revision

The manuscript is close to being suitable for publication, provided the above points—primarily related to language precision, figure clarity, and cautious interpretation—are addressed.

Sincerely,
Dr. Sepideh Asadi
