# Supplementary material for: Functionalized calcium carbonate microparticles in ethyl cellulose films: A vehicle for sustained amoxicillin release for medical applications
Source: PLoS One. 2026 Apr 2;21(4):e0320280. doi: 10.1371/journal.pone.0320280 (PMC13046161; doi:10.1371/journal.pone.0320280)
Supplement: Attachment — Submitted filename: Response to Reviewers 2025-05-27.docx [file pone.0320280.s011.docx]

**Reviewer #1**:

Dear Authors,
The research work is quite interesting with well problem statement but authors have designed the experiment for verifying material properties of amoxicillin loaded FCC not in a good manner and not thorough study to clearly understand the characteristic of the loaded FCCs. It is very difficult to follow mentioned experimental results step by step. It is very regret to recommend for rejection due to the quality of presented experimental data especially in the part of material characterization. For example:

The authors thank the Reviewer for the opinion regarding the worth of the work and acknowledge the aspects of concern. We hereby undertake to respond to the comments as follows:

1. To study the releasement of amoxicillin from FCC, FCC fines and FCC granules were used as carrier but to study the releasement of the amoxicillin from the Ethyl cellulose film, Omyapharm FCC500-OG was used instead without notifying the solid support to convince the possibility. Only particle size ranges of FCC fines and FCC granules were provided but not BET specific area not bulk density.

To clarify: the material family of functionalised calcium carbonate (FCC) retains consistent structural and chemical properties irrespective of sample name definition. We now have put more emphasis to this in the new manuscript text.

The use of a fractionated sample of FCC, in respect to fines and granules, was designed to study the impact of individual dispersed particle size versus structuration of those individual particles to form granules. The granular material has been shown earlier to accelerate advantageously pharmaceutical tablet disintegration in aqueous medium, for example, as illustrated in these extra references, which have now been added to the manuscript:

D. Preisig, *et al.*, ‘Drug Loading into Porous Calcium Carbonate Microparticles by Solvent Evaporation,’ *Eur. J. Pharm. Biopharm*. **87**, 548–558 (2014).

T. Stirnimann, *et al*., ‘Compaction of Functionalized Calcium Carbonate: A Porous and Crystalline Microparticulate Material with a Lamellar Surface,’ *Int. J. Pharm*. **466**(1–2), 266–275 (2014).

T. Stirnimann, *et al*., ‘Functionalized Calcium Carbonate as a Novel Pharmaceutical Excipient for the Preparation of Orally Dispersible Tablets,’ *Pharm. Res*. **30**(7), 1915–1925 (2013).

The context of exemplifying structuration of FCC in respect to release kinetic of amoxicillin is, therefore, undertaken here to establish if such structuration provides any release mechanistic advantages, stressing once again throughout that the particle structure and chemical nature of the particles remain intrinsically constant.

Omyapharm FCC500-OG illustrates the current commercially available product from which the experimental fractions were similarly derived. This text is now included in the manuscript.

Nitrogen adsorption analysis in terms of BET specific surface measurement and bulk density are now also reported.

2. The content of amoxicillin is not informed for releasement study from the loaded FCC granules (Figure 4) but from the loaded FCC fines, the releasement results is presented both from 15% and 30% amoxicillin loading. The releasement of neat FCC is not comparatively studied.

The loading fraction of amoxicillin was consistently 30 w/w% and 15 w/w% for all samples. Section 2.2. was unfortunately somewhat misleading in this respect as the term FCC particles was specifically used to refer generically to all sample forms. The text is now modified to confirm the loading across all FCC sample types. We understand the implication raised by the reviewer in that granulated material has an additional pore space, i.e. between the individual particles, into which the amoxicillin will also enter. This is now also expressed in the manuscript as follows:

[2.2. Particle Loading

To load amoxicillin into the porous FCC particles, the amoxicillin must first be dissolved in a

solvent with a low boiling point and associated high vapour pressure. This solution is then mixed with the FCC particles as present in all structural forms used in the study, namely FCC fines, granules and commercial Omyapharm, and the solvent is allowed to evaporate. During evaporation, amoxicillin precipitates both within the pores and on the surface of the FCC particles. In this study, a 3 g/L solution of amoxicillin in an ethanol/acetone mixture was prepared. This solution was then added to a predefined amount of particles (for 30 w/w% and 15 w/w % loading – 7 g FCC and 8.5 g FCC, respectively, per 1 L solution) and the solvent was evaporated using a rotary evaporator (30 °C and 400-150 mbar). It is recognised that the distribution of amoxicillin in the case of the granulated structure will include loading between particles, and not only on the surface, and within the pores of the individual particles. Given the nature of the granule concept, material between the particles, to a first approximation, is likely to behave similarly to that associated with the exterior of the particles, though it should be recognised that access of liquid to the interior of a granule may be hindered to reduced permeability. Such effects reveal themselves during the release experiment study.]

We appreciate the reviewer’s suggestion regarding the inclusion of a neat FCC control. However, as the release studies were based on UV-Vis spectroscopy at 272 nm—a wavelength specific to amoxicillin—neat FCC does not exhibit any absorbance at this wavelength. Therefore, it does not contribute to the measured signal, and its inclusion as a control would not affect the release data. This point is now clarified in the revised manuscript.

3. XRD diffraction pattern of precipitated amoxicillin is presented but its SEM micrograph is not shown, to verify different morphology.

The morphology of amoxicillin depends on two main factors, the dynamic of precipitation and the surface onto which the precipitate forms. Amoxicillin in free solution under slow evaporation leads to microscopic single crystals, however under induced rapid precipitation, such as by the presence of a surface or nucleation centres, naturally tends to be amorphous. When considering monomolecular layers, such as those deposited on the surface of the FCC particle pores, microscopic imaging, by definition, unfortunately fails to reveal such molecular structure information. It is for this reason that resort is made to XRD diffraction, as reported in the manuscript, it being sensitive to molecular and intermolecular structure of relevance here.

4. In the material characterization results from loaded FCC, the finding are rarely compared to other similar finding and made discussion.

We appreciate the Reviewer’s suggestion to broaden the contextual discussion of amoxicillin release from particulate systems beyond FCC, and we have now included comparative insights from other porous carrier systems. For example, Li et al. (2010) reported on organically modified MCM-41 mesoporous silica materials for amoxicillin delivery, where drug release was about 13% within 24 hours. Also, we reference studies such as Vafayi et al., who demonstrated a three-stage release profile from hollow silica nanospheres in simulated body fluid, and Bohns et al., who evaluated amoxicillin-loaded Eudragit S100 microparticles in dental applications. Their best formulation achieved a release of 16% amoxicillin within 96 hours. Additionally, Wang et al. who worked on the release of amoxicillin from Mg-HA porous particles. In this work 89% of the amoxicillin was released within 40hours. These examples have been incorporated into the revised manuscript to contextualize our results within the broader field of porous drug delivery systems. In contrast, our FCC-ethyl cellulose film composites demonstrate markedly extended release, supporting applications requiring long-term antimicrobial activity.

Li et al., *J. Colloid Interface Sci.*, 342 (2010), 607–613, https://doi.org/10.1016/j.jcis.2009.10.073.

L. Vafayi et al., Bioinorganic Chemistry and Applications 2013 Vol. 2013 Issue 1 Pages 541030 https://doi.org/10.1155/2013/541030

Bohns et al., Restor Dent Endod 2020 Vol. 45 Issue 4 Pages 50. DOI: 10.5395/rde.2020.45.e50

Wang et al., Materials and Manufacturing Processes 2022 Vol. 37 Issue 13 Pages 1500-1505 DOI: 10.1080/10426914.2021.2016824

**Reviewer #2**:

The study presents an interesting approach utilizing functionalized calcium carbonate (FCC) microparticles embedded in ethyl cellulose (EC) films for sustained antimicrobial drug release. This research is particularly relevant for indwelling medical applications, addressing the need for prolonged drug release in such devices.

The authors are grateful to the reviewer for recognising the value of the main target application for this work.

1. Please explain the novelty compared to published work in this field. Pure drug used or its salt used?

In many cases, pure drugs tend to express hydrophobicity due to their organic molecular constituents. The advantage of depositing a monomolecular layer is that the dynamic of this arrangement is constrained prior to exposure to water, and the rapid dissolution thereby prevents the macro-induced hydrophobicity. The citation regarding the delivery of nutraceuticals and flavourings (11) (Lundin Johnson *et al.*) illustrates this nicely. In the case of amoxicillin sodium salt dissolution is not normally impaired by any hydrophobic effects, and, as the Reviewer correctly suggests, loading on porous media is not a prerequisite to ensure dissolution. The target here is to retain a reservoir of the amoxicillin whilst at the same time supporting a continuous release of the active substance at a level above the minimum bacterial inhibition concentration to ensure a continuous antibacterial activity in applications prone to recurring infection.

2. The introduction can be improved with recent work in this filed, like, https://doi.org/10.1007/s12668-024-01722-3, https://doi.org/10.2174/0115748855274979231228103038,

https://doi.org/10.1007/978-981-99-1655-9_6,

https://doi.org/10.1016/j.foodhyd.2020.106206,

https://doi.org/10.1016/j.jdent.2024.105489,

https://journals.lww.com/jpbs/toc/2012/04001 etc

We thank the Reviewer for the suggested literature. We have carefully reviewed the references provided and acknowledge their contribution to the broader field of drug delivery. However, these works focus on different types of materials or delivery systems that do not closely align with the specific objective of our study—namely, the integration of drug-loaded FCC particles into ethyl cellulose films for sustained release. To maintain a focused and concise introduction that accurately reflects the scope of our work, we have decided not to include these particular references. Nevertheless, we appreciate the Reviewer’s effort in highlighting them.

4. No clarity on how formulation optimized?

The formulation *per se* has two interactive aspects, (i) the dissolution and rapid evaporation properties required when loading the porous medium particles, and (ii) the distribution of the material in and on the FCC particles and granulate structure. To meet the criteria for step (i), simple reduction of pressure using a rotovap sufficed. Meeting an optimum for (ii), the distribution of material, is much more complex, and indeed depends strongly on the dynamic of permeation and absorption of the amoxicillin solution into the porous medium, and the time progression for partial dissolution and precipitation/deposition within it. We agree with the Reviewer that further work would be needed to optimise the spatial distribution of the active within the porous medium. We now emphasise this limitation to the work in the text and suggest the need for further optimisation.

5. How, XRD, To understand the underlying mechanisms influencing amoxicillin release, give this understanding?

The Reviewer identifies an important link here between the XRD structure-related properties of the active agent, in this case amoxicillin, and the subsequent desired release into aqueous medium. The Reviewer will recognise the difficulty we faced, and discussed in some depth, regarding the likely structural nature of the active as deposited within the porous FCC particles. We had hoped that the evidence of molecular arrangement provided by XRD would be more convincingly displayed. Given the ambiguity the XRD data raised, we chose to be somewhat reserved in providing a detailed mechanism for the release. We agree this leaves some open questions, and resort to the experience shown by the nutraceutical and flavourings exercise reported earlier by Lundin-Johnson *et al*., especially noting the similar rapid early stage dissolution occurring during release from FCC in the absence of ethyl cellulose. We reference this difficulty in the manuscript and rely on future work revealing more clearly the specific mechanistic stages involved. Suffice it to say at this point that the controlled dynamic required for the proposed application has been reached despite the lack of a complete molecular-based mechanistic description. We trust this applications-focused result is of sufficient worth to support publication.

6. How author is sure that during casting in organic solvent, microparticle not dissolve/break?

Here we assume that the Reviewer is referring to the stability of the FCC structures. Given the inorganic structural nature of the FCC, we would not expect any microparticle dissolution or breakage. Furthermore, the work reported by

D. Preisig, *et al.*, ‘Drug Loading into Porous Calcium Carbonate Microparticles by Solvent Evaporation,’ *Eur. J. Pharm. Biopharm*. **87**, 548–558 (2014).

T. Stirnimann, *et al*., ‘Compaction of Functionalized Calcium Carbonate: A Porous and Crystalline Microparticulate Material with a Lamellar Surface,’ *Int. J. Pharm*. **466**(1–2), 266–275 (2014).

T. Stirnimann, *et al*., ‘Functionalized Calcium Carbonate as a Novel Pharmaceutical Excipient for the Preparation of Orally Dispersible Tablets,’ *Pharm. Res*. **30**(7), 1915–1925 (2013).

and now cited in the references, confirms the stability of the physical structures. The granulation is formed by the compression in close proximity of particle-particle adjacent surface platelets displaying van der Waals attraction, which, without inorganic breakdown, remains ubiquitous throughout any solvent processing. Only mechanical forces would be expected to disturb further the surface platelet structure.

7. Why HPMC K4m and HPMC K4M and Eudragit RS 100 selected as film former?

Here we assume that the Reviewer is referring to use ethyl cellulose (EC) as the film-forming polymer. We would like to clarify that HPMC K4M and Eudragit RS 100 were not part of our formulation work. The selection of EC was based on its well-established use in sustained-release systems, as it is a non-biodegradable yet biocompatible cellulose derivative widely studied for pharmaceutical encapsulation applications. EC is a biodurable polymer, and its molecular structure can be tuned through controlled degrees of substitution—specifically, the number of etherified hydroxyl groups per glucose unit—to achieve tailored solubility and viscosity profiles in various solvents. Moreover, although EC is not rapidly biodegradable under typical physiological conditions, cellulose and its derivatives are considered environmentally friendly materials, as they are ultimately degradable in natural ecosystems through microbial enzymatic activity (e.g., cellulases). This point is now explained in the revised manuscript.

We hope this clarifies the rationale for choosing EC as a film forming polymer.

8. The characterization of film like, Muco adhesion, tensile strength, swelling etc. should be compiled.

Here the Reviewer takes the work forward into the application environment in respect to both mechanical properties and properties in-vivo. We can but agree wholeheartedly that strength, swelling properties and surface interaction of the ethyl cellulose construct with surrounding mucous membranes is of crucial importance. Simply said, we have not moved on to the important applications environment at this stage and such questions are left open. We now draw attention to this next analytical step in the manuscript.

Reviewer #3:

Comment 1: Keep the term fixed either extended or sustained release system.

Thank you for raising this word-meaning point. We have reviewed the use of the two terms and made sure that they are not used synonymously. They do represent different effects: “sustained” aims to maintain a constant drug concentration (level plateau-like release) over a period, whereas “extended” refers to maintained therapeutic drug levels for an extended duration. We have now added this clarification of definition between the two words - subtle but important.

Comment 2: Can you check this line ‘an increased release rate from the particles compared to pure amoxicillin powder’ release rate will be extended? And also make it which one is considered here a particle?

Thank you for drawing attention to the potential ambiguity here. We have rephrased to read:

‘an increased release rate from the porous FCC particles compared to direct dissolution of pure amoxicillin powder’

Comment 3: ‘sustained drug delivery acts to release the drug at a controlled rate’ There is a difference between sustained and controlled release

Yes, indeed, we differentiate between rate of release and sustained release. Once again this lies closely connected to the response to Comment 1, however we agree that the phrase is not correct in terms of cause and effect:

The target is to achieve sustained release, i.e. a plateau-like release rate so as to maintain the minimal bacterial inhibitory concentration, and to meet this target for a given loading the rate of release from a sufficient store must be correct to provide the sustained effect. So, we reword as follows:

‘sustained drug delivery requires a specific controlled release rate dependent on the loading level of active on, and the dynamic of release from, the porous medium’

Comment 4: significant efforts are directed at developing new indwelling urinary catheters (IUCs)’ discuss related works on this. See such articles Int J Biol Macromol, 162, 2020, 1849-1861. Polymers. 2022; 14 (4): 746; J. Drug. Deliv. Sci. Tech., 72, 2022, 103380

We thank the Reviewer for providing further citation material. Reference discussion to this additional work is now included.

Comment 5: mention the molecular weight of EC and the subsection process is not proper.

We thank the Reviewer for this comment. The molecular weight of ethyl cellulose (EC) grade N100 is not explicitly specified by the manufacturer since EC is a polymer with a distribution of molecular weights. However, the viscosity of EC N100, typically measured as 100 mPa·s for a 5% solution in toluene/ethanol (80:20) at 25 °C, serves as an indirect indicator of its molecular weight. This information has now been included in the revised manuscript.

We assume the Reviewer is referring to the organization of the subsections within the *Materials and Methods* section. We thank the Reviewer for raising the point regarding the subsection structure, which may have appeared unclear. To address this, we have revised the order and structure of the subsections to improve clarity and readability. We have also ensured consistent terminology when referring to FCC in its different structural forms (e.g., fines, granules, or Omyapharm), and clarified which material was used at each step of the process. We hope this restructuring resolves the confusion and better reflects the experimental workflow.

Comment 6: the amoxicillin must first be dissolved in a solvent with a low boiling point. ‘Mention the solvent

We thank the Reviewer for this comment. We would like to clarify that amoxicillin was dissolved in a mixture of ethanol and acetone, both of which are low-boiling-point solvents commonly used in pharmaceutical formulation. This information is already included in the *Materials and Methods* section of the manuscript, but we have now ensured it is clearly stated and easy to locate for the reader.

Comment 7: amoxicillin release in water was determined using a thermogravimetric analyzer’ This procedure is not proper. Please cite some related works if anyone has followed the method.

We thank the Reviewer for this important comment. While thermogravimetric analysis (TGA) is not traditionally used to *directly* measure drug concentration in solution, in our study it was used indirectly to quantify the remaining amoxicillin in the FCC particles after aqueous release, allowing the amount released to be inferred by mass balance.

Rationale for using TGA**:**
TGA measures weight changes in a sample as it is heated, allowing for the detection of components that volatilize at different temperatures. In our context:

- Organic Material in Inorganic Matrix**:** Amoxicillin, an organic compound, decomposes at a lower temperature compared to the inorganic FCC matrix. This difference enables the quantification of amoxicillin content based on weight loss observed during TGA.
- Post-Release Analysis**:** After the release study, the FCC particles were separated by centrifugation, and the collected FCC cake was analyzed using TGA. The weight loss corresponding to the decomposition of amoxicillin provided an estimate of the drug retained in the particles, thereby allowing us to infer the amount released into the aqueous medium.

To support the validity of this approach, TGA has been effectively used in related contexts to quantify drug loading in various systems:

- Mesoporous silica nanoparticles with drug detection down to 0.77% (w/w) <http://dx.doi.org/10.2139/ssrn.4355731>
- Diatom silica microparticles for assessing drug content based on surface modification. <https://doi.org/10.1016/j.ijpharm.2012.12.012>
- PLGA microspheres, where oxaliplatin content was quantified

<https://doi.org/10.1002/masy.201500181>

- Amorphous mesoporous titania (APMT) nanoparticles carvedilol loading <https://doi.org/10.1016/j.micromeso.2011.12.013>

These examples show that TGA can be used to evaluate drug content on a wide range of systems. We have now added clarifying details and references in the *Materials and Methods* section to better explain how TGA was used.

Comment 8: If XRD procedure is needed in so details

We recognise that the discussion surrounding the results from XRD and the method used is extensive. We justify this due to the problem of defining the structural form of the active material when loaded into the porous particles, which we consider crucial to a further understanding of the release mechanisms involved.

Comment 9: Calibration curve can be added

We thank the Reviewer for this helpful suggestion. The UV-Vis calibration curve used to quantify amoxicillin concentration at 272 nm has now been included in the Supplementary Information (Figure S1). The calibration curve follows a standard linear response within the tested concentration range and agrees with previously reported results for amoxicillin quantification in aqueous media.

https://doi.org/10.1155/2020/5860487

This approach is commonly used in related studies.

Comment 10: Please check Figure 5

In asking about checking the content of Figure 5, the Reviewer has indeed recognised the problematics, mentioned above, in determining the physical state of matter of the loaded active amoxicillin. The amoxicillin sample in its natural powder form displays its complex crystallinity. The inorganic host porous particles demonstrate their inorganic crystal structure, however the loaded amoxicillin shows neither crystalline peaks nor an amorphous background intensity in addition to those associated with the inorganic host particles alone.

The authors extend their thanks once again, and recognise that via the responses to the Reviewers’ comments, certain aspects can be better clarified and the paper, as result, enhanced.
