# Supplementary material for: Functionalized calcium carbonate microparticles in ethyl cellulose films: A vehicle for sustained amoxicillin release for medical applications
Source: PLoS One. 2026 Apr 2;21(4):e0320280. doi: 10.1371/journal.pone.0320280 (PMC13046161; doi:10.1371/journal.pone.0320280)
Supplement: Attachment — Submitted filename: Review Comments-2.docx [file pone.0320280.s009.docx]

**Functionalized Calcium Carbonate Microparticles in Ethyl Cellulose Films: A Vehicle for Sustained Amoxicillin Release for Medical Applications.**

The manuscript explores the incorporation of amoxicillin-loaded functionalized calcium carbonate (FCC) microparticles into ethyl cellulose (EC) films for sustained antibiotic delivery. While the research topic is of significant relevance and potential impact, several critical concerns regarding the experimental design, methodological detail, data presentation, and interpretation need to be addressed to enhance its scientific rigor and clarity:

**1. Lack of Detailed Methodology:** The manuscript offers insufficient information about the experimental procedures particularly for drug loading, film fabrication, and release studies. For the work to be reproducible and validated, the authors should specify detailed protocols, including solvent types and volumes, concentrations, incubation conditions, processing times, and drying procedures.

- The "solvent evaporation method" used for amoxicillin loading into FCC is roughly described. Precise details such as the solvent employed? the volume use? the incubation duration, temperature, agitation, and how the loading efficiency was quantified are essential. Explicitly justifying the choice of this method in the context of optimizing drug loading and stability would be beneficial.
- Similarly, the film casting process lacks information parameters like solvent choice? drying conditions? and resulting film thickness should be detailed to ensure reproducibility.

**2. Analytical Instruments and Techniques:** While SEM and XRD are mentioned for characterization, the specific models, configurations, operational parameters, and the conditions under which analyses were performed are not provided.

- For drug release evaluation, the authors should specify the apparatus used? the medium composition? temperature, agitation speed, and sampling protocol.
- Clarifying these details is critical to assess the reliability, reproducibility, and comparability of the release data.
- Regarding UV-vis spectrometry, details such as the calibration curve, detection wavelength, detection limits, and how the drug concentration was derived should be included.

**3. Justification for the Release Study Method:**

- Clarifying the rationale behind the selected method, including its advantages (e.g., mimicking physiological conditions, controlling variables) and limitations, will strengthen the credibility of the release data.

**4. Assessment of Drug Release and Antibacterial Activity:**

 The manuscript reports increased release rates based on UV-vis data but omits details such as the calibration procedure, wavelength selection, and detection limits.

- The underlying release mechanism diffusioncontrolled, erosion-controlled, or a combination—remains unexplored. Incorporating kinetic modelling (e.g., zero-order, first-order, Higuchi) would provide valuable mechanistic insights.
- The antibacterial efficacy claim lacks methodological description: specify bacterial strains (e.g., *E. coli*, *S. aureus*), assay types (e.g., agar diffusion, broth dilution), controls used, and results (zone of inhibition, MIC values).
- Such data are vital to substantiate the system’s functional efficacy.

**5. Data Presentation and Figures:** The micrographs and diffraction patterns presented are of limited clarity and annotation.

- High-resolution images with clear labels indicating features such as the distribution of amoxicillin within FCC particles, porosity, and particle morphology are necessary.
- Quantitative data should accompany figures

**6. Discussion and Contextualization:** The manuscript lacks an in-depth discussion linking the findings to existing literature and elucidating how this system offers advantages over current drug delivery platforms.

- Particular attention should be given to the long-term release profile, the mechanisms controlling drug release, and the potential clinical applications.
- Addressing limitations, such as potential stability issues or scalability, would also provide a balanced perspective.
