# Supplementary material for: Functionalized calcium carbonate microparticles in ethyl cellulose films: A vehicle for sustained amoxicillin release for medical applications
Source: PLoS One. 2026 Apr 2;21(4):e0320280. doi: 10.1371/journal.pone.0320280 (PMC13046161; doi:10.1371/journal.pone.0320280)
Supplement: Attachment — Submitted filename: Response to Reviewers_FCC-EC Sustained Release.docx [file pone.0320280.s013.docx]

Reviewer #1: Dear Authors,

Thank you for the revisions made according to the reviewer's comments. Some revision has been done well. However please fulfill these following comments;
1. In Fig 4. Please give information on the amoxicillin loading for loaded FCC granules as shown in blue squares.

We thank the reviewer for noting that the loading level for the FCC granules in Fig 4 was not clearly stated. The granules shown in blue squares correspond to 30 wt % amoxicillin loading, as confirmed by TGA. This information has now been added to the figure caption and clarified in the Results section.

2. If possible, please provide the UV spectra of FCC fines, FCC granules and, Omyapharm particles (used to prepare EC film, in a range that cover wavelength of 272 nm as Data Availability.

We thank the reviewer for the suggestion. FCC fines, FCC granules, and Omyapharm consist solely of inorganic calcium carbonate–calcium phosphate materials, which do not absorb in the UV region near 272 nm. To confirm this, we have now added UV–Vis spectra of these unloaded materials (range 220–300 nm) in the Supplementary Information (S3 Fig), demonstrating no measurable absorbance at 272 nm. This supports the validity of using this wavelength for amoxicillin quantification.

3. Please workout more on the authors' response to reviewer's comment 3 on the aspect of molecular structure of the precipitated amoxicillin and intermolecular structure. In addition, the image of shown in Fig 7 (C) does not help to make relationship between the sustained release of precipitated amoxicillin from FCC. Not only the molecular and intermolecular structure affects the releasement performance but also the physical characteristics of the precipitated of amoxicillin inside the FCC and on the surface.

We thank the reviewer for emphasizing the need to more clearly relate molecular and physical structure to release behaviour. We have now expanded the discussion in Results and Discussion Scanning Electron Microscopy Section to describe how the confined amorphous amoxicillin within FCC pores and the nanocrystalline surface deposits together determine the observed two-stage release. The SEM (Fig. 7) and XRD data are now interpreted jointly to link structural form and release kinetics.

Reviewer #2: The author has successfully addressed all the comments and suggestions provided in the previous review. The revisions and editions made are satisfactory and have enhanced the overall quality of the manuscript.

We sincerely thank the reviewer for the positive feedback and for acknowledging the improvements made in the revised manuscript. We appreciate the reviewer’s careful evaluation and supportive recommendation.

Reviewer #3: The paper can be accepted. The comments have been addressed. The author has responded point to point comments

We thank the reviewer for the encouraging comments and are pleased that the revisions satisfactorily addressed all previous points. We appreciate the recommendation for acceptance.

Reviewer #4: This study investigates FCC microparticles integrated in ethyl cellulose films for sustained amoxicillin delivery—an innovative and clinically relevant concept targeting catheter-related infections. The combined use of SEM, UV-vis, synchrotron XRD, and TGA strengthens the material analysis. Nonetheless, the manuscript currently falls short in methodological completeness, clarity, and novelty justification

Major Issues (Essential for Revision):
1. Material transitions and clarity
o The manuscript frequently switches between FCC fines, granules, and Omyapharm (e.g., in Chapters 2 and 3). A comparative table listing surface area (BET), morphology, rationale, and selection criteria for each form would greatly improve clarity.

We thank the reviewer for this valuable suggestion. To improve clarity and transparency regarding the use of the different FCC materials, we have now included a comparative table (Table 1) in the *Materials and Methods* section. This table summarizes the key physicochemical parameters—particle size range, BET surface area, bulk density, and morphology—for FCC fines, FCC granules, and Omyapharm FCC500-OG, as well as their specific experimental roles and rationale for selection.

We have also simplified the corresponding text to introduce the FCC material family and to guide the reader to Table 1, thereby reducing redundancy while improving readability. This addition clarifies the relationships between the various FCC forms and supports a coherent understanding of their use throughout the study.

2. Film characterization for application
o Films designed for indwelling use require mechanical (tensile strength), swelling, and mucoadhesion data. At minimum, quantitative swelling or tensile information should be included, or limitations acknowledged.

We thank the reviewer for highlighting this important point. The present study was designed as a *proof-of-concept* investigation to demonstrate the feasibility of using amoxicillin-loaded FCC particles embedded in ethyl cellulose (EC) films as a platform for achieving sustained antibiotic release over extended periods. Consequently, our primary focus was on establishing and validating the release mechanism rather than on the full mechanical or mucoadhesive characterization of the films.

We fully agree that such properties—tensile strength, swelling behavior, and mucoadhesion—are essential for future translation of the material system to indwelling or implantable applications. We have now added a statement in the *Discussion* section explicitly acknowledging this limitation and noting that comprehensive mechanical and interfacial property assessments will be part of our future work aimed at optimizing the FCC–EC film system for clinical device coatings.

3. Drug release quantification
o Relying on TGA for drug release estimation is unconventional. While UV vis release curves are present, detailed release kinetics (e.g., cumulative release, release rate ± SD, model fitting) must be shown. Clarify that TGA data are supplementary and residual only.

We thank the reviewer for this insightful comment. We agree that thermogravimetric analysis (TGA) is not conventionally used as a primary quantification method for drug release. As discussed in our earlier response, TGA in this study was used solely as a **complementary technique** to confirm **residual amoxicillin content** in the FCC particles after release and to validate the UV–Vis–based release data. We have now clarified this in the *Materials and Methods* section and in the *Results* text, explicitly stating that TGA results serve as **supplementary confirmation** only.

Regarding release kinetics, we acknowledge that the current study focuses on demonstrating proof-of-concept sustained release rather than full kinetic modeling. Because the release experiments extended over several months, replicate measurements under identical conditions were not feasible within this work. However, we have included an additional dataset for a thinner FCC-loaded EC film (160 µm), which shows a very similar long-term biphasic release profile—an initial burst followed by a slower sustained phase (see new Supplementary Information S4 Fig). This supports the reproducibility and robustness of the observed release behavior across different film thicknesses. Although a corresponding reference film containing the physical amoxicillin–FCC mixture at 160 µm was not available, this additional result reinforces the proof-of-concept findings. Future studies will include statistical replication and kinetic modeling to further quantify the release mechanism.

4. Novelty and comparative context
o Similar systems (e.g., Mg HA, Eudragit, MCM 41) have been used for amoxicillin delivery. The manuscript should explicitly highlight what is novel—such as use of FCC in EC films—and compare performance metrics (release duration, loading capacity) to existing systems.

We thank the reviewer for this helpful suggestion. We have now revised the *Discussion* sections to explicitly emphasize the novelty of this work and to provide a direct comparison with reported amoxicillin delivery systems. The key innovation lies in the use of **functionalized calcium carbonate (FCC)**—a biocompatible, porous inorganic carrier—**embedded within a biodurable ethyl cellulose (EC) matrix** to create a composite film enabling long-term, sustained antibiotic release. To our knowledge, this is the first demonstration of FCC integrated into EC films for controlled antimicrobial release.

We have also included a brief comparative summary of representative systems, noting that:

- Amoxicillin release from **MCM-41 mesoporous silica** reached ~13 % within 24 h (Li et al., 2010).
- **Eudragit S100** microparticles achieved 16 % release over 96 h (Bohns et al., 2020).
- **Mg–HA porous particles** released ~89 % of the drug within 40 h (Wang et al., 2022).

In contrast, the **FCC–EC films reported here maintain measurable amoxicillin release over more than four months**, while the film structure remains intact and stable in the release medium. This markedly longer release duration underscores the novelty and potential of the FCC–EC approach for indwelling medical coatings.

5. Terminology consistency
o Use “sustained” exclusively throughout. Currently, the manuscript alternates between “sustained” and “extended.” Ensure consistent usage.

We thank the reviewer for pointing out the inconsistency in terminology. The manuscript has now been carefully revised to use the term **“sustained release”** consistently throughout, as it best reflects the controlled and prolonged release behaviour observed for the FCC–EC film system.
Minor Suggestions:
• Correct remaining typographical and formatting issues (e.g., “Formatted: Font: 16?pt”).
• Expand figure legends (e.g., labeling apparatus components in Figure?2).
• Define all solvents and abbreviations in the Methods section first mention.
• Clarify that ethyl cellulose and FCC are biocompatible and sterile, as relevant to medical application.

The study is innovative and scientifically sound, but essential methodological work and clarity improvements are necessary for full publication. I encourage the authors to address these points comprehensively.

We thank the reviewer for these helpful editorial and clarity suggestions. The following corrections and additions have been made in the revised manuscript:

1. **Typographical and formatting corrections:** All remaining formatting artifacts (e.g., “Formatted: Font: 16 pt”) and minor typographical errors have been carefully removed and corrected throughout the document.
2. **Expanded figure legends:** Figure legends have been revised for clarity. In particular, **Figure 2** now includes a brief description of the experimental setup.
3. **Definition of solvents and abbreviations:** All solvents, materials, and abbreviations are now defined at their first mention in the *Materials and Methods* section to ensure clarity for readers less familiar with the terminology.
4. **Biocompatibility and sterility clarification:** Additional text has been added in the *Materials and Methods* section noting that **ethyl cellulose (EC)** and **functionalized calcium carbonate (FCC)** are recognized as **biocompatible materials** commonly used in pharmaceutical and biomedical applications. We also note that the materials and films were handled under clean laboratory conditions; however, **sterility testing** was beyond the scope of this proof-of-concept study and will be addressed in future work aimed at medical device translation.

Reviewer #5: I recommend that the authors substantially revise the manuscript to include detailed methodologies, robust characterization data, well-annotated images, and thorough discussions. Doing so will significantly improve the clarity, reproducibility, and scientific impact of the work.

We appreciate the reviewer’s valuable and constructive feedback. In the revised manuscript, we have thoroughly addressed each of these points:

- **Detailed methodologies:** The *Materials and Methods* section has been extensively expanded to include precise solvent compositions, concentrations, equipment parameters, and experimental configurations (e.g., film preparation, FCC loading, and release testing setup). These revisions greatly improve the transparency and reproducibility of experimental procedures.
- **Robust characterization data:** Additional data and clarifications have been incorporated. Specifically, Table 1 now summarizes the physicochemical parameters (surface area, morphology, bulk density, and rationale) of all FCC materials. The UV–Vis calibration curve for amoxicillin quantification is included in the *Supporting Information*, together with new UV–Vis spectra of unloaded FCC fines, FCC granules, and Omyapharm particles showing no absorbance near 272 nm. This confirms that the release measurements originate solely from amoxicillin.
- **Well-annotated images:** Figures 2, 6, and 7 have been revised with expanded legends and clear labeling of all components and morphological features, improving interpretability.
- **Thorough discussion:** The *Results and Discussion* section has been strengthened. The relationship between amoxicillin’s structural state (amorphous/nanocrystalline) and its release profile is now discussed in detail, and the novelty of the FCC–EC system is explicitly compared with other amoxicillin delivery platforms (e.g., Mg–HA, MCM-41, Eudragit).

Together, these revisions improve the manuscript’s clarity, reproducibility, and scientific impact, fully in line with the reviewer’s recommendations.

Reviewer #6: I can recommend publishing, however some the authors need to answer the following question and accordingly revise the manuscript.
1) How did the authors determine crystal unit cell parameters from a bench-top XRAY machine. Did they index the powder pattern? If so, the results should be documented? If they grew single crystal, then did the simulated pattern from single crystal match the experimental pattern ? Has the structure been uploaded to the CCDC database? These details are missing and important to understand how these results were obtained.

We thank reviewer for raising this relevant point. Crystal unit cell parameters for the pure crystalline amoxicillin powder were obtained by indexing the powder diffraction patterns obtained with the benchtop diffractometer using HighScore, Malvern Panalytical's software. As the resulting parameters are in good agreement with published work [17-19], no further simulation was run. Details of amoxicillin's crystal structure are publicly available in these referred works [17-19]. The text of the manuscript has now been updated to clarify this better, including by explicitly mentioning the use of the HighScore software.

2) For the different dissolution experiments the authors have not described whether the tests were conducted under sink or non-sink conditions? If under sink conditions, how will this correlate to drug release from catheter once implanted?

We thank the reviewer for this important question and for the opportunity to clarify. The dissolution and release experiments were conducted under **different hydrodynamic conditions** depending on the material system:

- **FCC particle release studies** were performed under **sink conditions**, ensuring that the concentration of amoxicillin in the release medium remained well below its solubility limit throughout the experiment. This allowed assessment of the intrinsic release kinetics from the porous carrier without limitations from solution saturation.
- **Film release studies** were conducted under **non-sink conditions**, as the intent was to simulate a diffusion-limited environment more representative of an **in vivo indwelling surface**, such as a catheter coating. In such settings, local fluid exchange is restricted, and maintaining sink conditions is neither realistic nor physiologically relevant.

We have now clarified these experimental conditions in the *Materials and Methods* section and added a short explanatory note in the *Discussion* to emphasize that the non-sink release behavior of the films corresponds to the diffusion-controlled antibiotic release expected in practical device applications.

3) Did the authors do a mass balance experiment and confirm that at the end of 5 month dissolution experiments the amoxicillin loaded in Omyapharm had the remaining drug in the intact in the films or had the drug degraded by that time?

We thank the reviewer for this important question. A full mass balance study and degradation analysis were not performed within the present proof-of-concept investigation. The primary goal of this work was to demonstrate the feasibility and long-term release behavior of amoxicillin from the FCC–EC composite films. Although the total released and residual drug contents were estimated by UV–Vis and TGA, we did not specifically evaluate the chemical stability or degradation profile of amoxicillin over the extended 5-month period. We fully agree that assessing the integrity of the released and residual amoxicillin is essential for future work.

4) There is a lack of statistical data in the dissolution release models? No error bars seen and std deviation mentioned in the experiments.

We thank the reviewer for highlighting this important point. As noted in our previous responses, the dissolution and long-term release experiments were conducted as **single-sample, proof-of-concept studies** designed to demonstrate the feasibility and temporal characteristics of the FCC–EC sustained-release system. Because each experiment extended over several months, replicates under identical conditions could not be practically completed within the scope of this work. Consequently, error bars and standard deviation values are not presented. We fully recognize that statistical validation is crucial for quantitative kinetic modeling and will be included in future work.
