# Supplementary material for: Functionalized calcium carbonate microparticles in ethyl cellulose films: A vehicle for sustained amoxicillin release for medical applications
Source: PLoS One. 2026 Apr 2;21(4):e0320280. doi: 10.1371/journal.pone.0320280 (PMC13046161; doi:10.1371/journal.pone.0320280)
Supplement: Attachment — Submitted filename: Review comments-2 (2).docx [file pone.0320280.s012.docx]

**Functionalized Calcium Carbonate Microparticles in Ethyl Cellulose Films: A Vehicle for Sustained Amoxicillin Release for Medical Applications**

The manuscript presents a scientifically sound and innovative approach for sustained amoxicillin release using functionalized calcium carbonate (FCC) microparticles embedded in ethyl cellulose (EC) films, targeting indwelling medical device applications (e.g., catheters).

**Strengths**

- The methodology is well described, including precise experimental details and advanced characterization (SEM, XRD, UV-vis, TGA).
- The composite system demonstrates a biphasic and prolonged release profile, maintaining drug levels above MIC for over five months, which is notably longer than most comparator systems (e.g., Mg-HA, MCM-41, Eudragit).
- The novelty of integrating biocompatible FCC with EC matrix for extended antimicrobial release is clearly justified with comparative literature context.

**Weaknesses**

- The long-term dissolution/release studies lack statistical replication or error analysis due to single-sample, proof-of-concept design; quantitative kinetic modelling is not possible from this dataset.
- Mechanical properties relevant to indwelling applications (tensile strength, swelling, mucoadhesion) are not evaluated and should be addressed in future studies.
