# Supplementary material for: Functionalized calcium carbonate microparticles in ethyl cellulose films: A vehicle for sustained amoxicillin release for medical applications
Source: PLoS One. 2026 Apr 2;21(4):e0320280. doi: 10.1371/journal.pone.0320280 (PMC13046161; doi:10.1371/journal.pone.0320280)
Supplement: Attachment — Submitted filename: Response to Reviewers.docx [file pone.0320280.s015.docx]

**Response to Reviewers**:

**Reviewer #1**: Dear Author,

The revisions are fully fulfilled to all points of previous comments and suggestions.

**Response to Reviewer #1**: We sincerely thank the reviewer for the positive and encouraging assessment of our revised manuscript. We are pleased that all previous comments were satisfactorily addressed, and we appreciate the reviewer’s supportive recommendation.

**Reviewer #4**: Thank you for the thoughtful revision. The manuscript is much improved in clarity and positioning. I appreciate the addition of the FCC materials comparison (particle size/BET/bulk density with usage rationale), the clearer description of sink (particles) vs non-sink (films) conditions, the inclusion of UV-vis controls showing no FCC absorbance at 272 nm, and the expanded comparison to prior amoxicillin delivery systems. The sustained multi-month release under non-sink conditions is a useful proof-of-concept for indwelling applications.

**Recommendation**: Minor Revision

**What has been satisfactorily addressed**

Materials clarity: Table summarizing fines, granules, and Omyapharm with BET/density and rationale greatly improves readability.

Terminology: Consistent, correct use of “sustained release” with a brief definition.

Context/novelty: Stronger discussion vs MCM-41, Mg-HA, Eudragit, hydrogels, hollow silica; the FCC-in-EC approach and months-long profile are now well framed.

Analytical specificity: UV spectra for FCC materials confirm no absorbance at 272 nm, supporting the release measurements.

**Requests before acceptance (minor but important)**

**Statistics & replication for release data**.
Please report the number of independent replicates for particle (Figs 3–4) and film (Figs 8–9) release experiments. Where feasible, provide mean ± SD and indicate n in figure captions. If the 5-month film curve was run on a single representative sample (as stated), keep it as proof-of-concept but clearly label it as such in the text and captions.

**Basic kinetic analysis**.
Add simple model fits (e.g., Higuchi and/or Korsmeyer–Peppas) to the film data—separating the initial burst and the longer sustained phase is fine. A small SI table with parameters (and R², n for Korsmeyer–Peppas) is sufficient. If you believe non-sink conditions make a specific model inapplicable, a brief rationale will suffice.

**End-point mass balance for films**.
Provide an assay at the end of the 5-month study confirming remaining drug in the films and commenting on degradation. TGA (as used for particles) is acceptable; HPLC/LC-MS would be ideal if available. This will strengthen the long-duration result.

**Compact methods summary**.
Include a short table (main text or SI) listing, for each experiment: matrix (particles/films), sink vs non-sink, total volume, temperature, agitation/flow, exposed surface area (your coiled film geometry is clear—just recap the calculated area). This will improve reproducibility.

**XRD reporting clarity**.
Please clarify whether the powder pattern was indexed and temper any structural claims that would require single-crystal data. A one-sentence note is enough; a CCDC deposition is not necessary here.

**Limitations statement (applications**).
Keep/strengthen the explicit note that tensile strength, swelling, and mucoadhesion of EC films were not examined in this proof-of-concept and are planned for future work toward indwelling use.

**Minor edits**

Remove any residual tracked-changes artifacts (e.g., “Formatted: …”).

Keep explicit loading information in Fig. 4 caption.

If you add statistics/kinetic fits/mass-balance data to SI, please reference them in the Data Availability statement and relevant figure captions.

With the above clarifications and small additions, the manuscript will meet PLOS ONE’s rigor and transparency standards.

**Response to Reviewer #4**

**Statistics & Replication for Release Data**

We thank the reviewer for highlighting the importance of clearly stating the level of replication for the release experiments.

Figs. 3 and 4 (particle release experiments): These release experiments were performed multiple times; however, they were carried out under slightly different experimental conditions (e.g., differing water volumes, acquisition time settings, and hydrodynamic parameters). Because these variations influence the release rates, the resulting datasets cannot be combined to generate statistically meaningful *mean ± SD* values. For this reason, we present the curves from a single representative experiment for each condition, and we now explicitly state this in both the text and the corresponding figure captions.

Figs. 8 and 9 (film release experiments): The long-term film release data were obtained from a single representative sample, as noted earlier. Given the 5-month duration of these measurements, replication under identical conditions was not feasible within this proof-of-concept study. We agree that this should be clearly communicated, and we have now revised the text and figure captions to explicitly indicate that the results derive from a single measurement.

**Basic Kinetic Analysis**

We thank the reviewer for the thoughtful suggestion to include simple kinetic model fits (e.g., Higuchi or Korsmeyer–Peppas). We agree that such models are valuable tools for interpreting controlled-release behavior. However, applying these classical kinetics to our system is not directly appropriate for the following reasons:

1. **The film release experiments were conducted under non-sink conditions**, intentionally chosen to simulate the diffusion-limited environment relevant to indwelling medical devices. Classical models such as **Higuchi** and **Korsmeyer–Peppas** assume sink conditions and constant diffusivity. Under non-sink conditions, the concentration build-up in the release medium alters the driving force for diffusion, violating key model assumptions and leading to unreliable or non-physical parameter estimates.
2. **The data exhibits a biphasic profile** consisting of i) an initial burst phase driven mainly by surface-accessible amoxicillin, followed by ii) a much slower, months-long sustained phase controlled by water penetration into the EC matrix and dissolution from FCC domains.
   These phases arise from distinct mechanisms, meaning that a single-regime model would not capture the entire curve meaningfully.
3. **Statistical model fitting is not robust due to the single-sample nature of the long-term film experiment.** With only one extended-duration dataset, regression-based extraction of kinetic parameters would not yield statistically defensible values.

For these reasons, rigorous application of Higuchi or Korsmeyer–Peppas kinetics would risk over-interpretation and may mislead the reader. Instead, we now include in Supporting Information a short explanatory note outlining why classical kinetic modeling is not applicable to the present non-sink, long-term dataset. We believe this provides the necessary transparency while avoiding the introduction of unreliable quantitative parameters.

We fully agree that kinetic modeling will be essential in future studies and will be incorporated once replicated, sink-controlled experiments are performed under well-defined conditions.

**End-Point Mass Balance for Films**

We thank the reviewer for this valuable suggestion and fully agree that an end-point mass balance confirming the remaining amoxicillin content would further strengthen the long-term release data. Unfortunately, it is not possible to perform a reliable post-hoc assay on the original film samples. These specific samples have been stored at ambient laboratory conditions for several years since the completion of the long-term experiment, and any residual amoxicillin may have undergone degradation during this extended storage period. Conducting TGA or HPLC on these aged samples would therefore not reflect the true chemical state of the films at the 5-month time point (distinguish between drug released during the experiment and drug degraded during storage) and could lead to misleading conclusions.

We fully recognize that end-point drug quantification and degradation assessment are essential for future development of this system. In future work, we will ensure to record replicated long-term release studies in parallel with TGA and HPLC/LC-MS assays at predefined time points to establish full mass-balance and stability profiles.

**Compact Methods Summary Table**

We thank the reviewer for this helpful suggestion to improve clarity and reproducibility. In response, we have now added a concise summary table in the Supporting Information (referred in the Materials and Methods section) that lists the key methodological parameters for each experiment, including: sample type (particles or films), sink vs. non-sink conditions, release medium volume, temperature, agitation or flow settings, and the calculated exposed surface area for the coiled-film geometry. This table consolidates information that was previously distributed across several sections and should greatly facilitate reproducibility and comparison of experimental conditions.

**XRD Reporting Clarity**

We thank the reviewer for pointing out the need to further clarify the XRD analysis and to temper structural claims. In the revised manuscript, we have now explicitly stated that the powder diffraction patterns from the benchtop diffractometer were indexed and refined using the HighScore software (Malvern Panalytical). We also clarified that the extracted unit cell parameters—although obtained from powder data—are in close agreement with reported orthorhombic single-crystal structures from the literature. This wording ensures that no interpretation is implied beyond what powder diffraction supports, and no single-crystal level claims are made.

For the synchrotron nano-XRD experiments, we emphasize again that the analysis is qualitative in nature. The manuscript already notes that the probed area is very small and that the results are used only to differentiate amorphous, nanocrystalline, or mesophase-like domains rather than to derive quantitative structural parameters. The revised text now highlights this explicitly to avoid any misunderstanding regarding the scope and limitations of the nano-XRD interpretation.

Together, these clarifications address the reviewer’s concern and ensure that all structural conclusions remain aligned with the capabilities and limitations of powder and nanoscale XRD methods.

**Minor Edits**

We thank the reviewer for these helpful final suggestions. All remaining tracked-changes artifacts have now been removed throughout the manuscript. We confirm that the explicit loading information (“30 wt% amoxicillin-loaded FCC granules”) has been retained in the Fig. 4 caption as requested. The additional materials added to the Supporting Information—including the methodological summary table and explanatory note on kinetic modeling—are now referenced in the Data Availability statement.

Reviewer #5: (No Response)

Reviewer #6: I support publication, but I would like the authors to clarify the following before acceptance.
1) We can speculate that the driving mechanism for nanocrystal formation could be related to a sorption effect of molecules in contact with a surface which could provide a nanoscale molecular orientation effect, a conformation that prevents the lowest energy intermolecular structure, namely preventing crystal formation.

• Could the authors clarify "lowest energy intermolecular structure"? Amorphous amoxicillin suggests a high-energy metastable state, while the crystalline form represents a low-energy conformation.

2) Did the authors examine, or can they propose, whether amoxicillin underwent crystallization during the release studies? This consideration could potentially account for the observed release profile in relation to the differences in solubility between crystalline and amorphous forms of amoxicillin.

3) How does the solubility of amorphous amoxicillin compare to crystalline amoxicillin? How can changes in crystal form during formulation affect the product's performance and release?

**Response to Reviewer #6**

1) We thank the reviewer for the opportunity to clarify our meaning. In our original wording, “lowest energy intermolecular structure” referred to the thermodynamically most stable crystalline arrangement attainable *under the specific boundary conditions inside the FCC particles*, not to the global bulk crystalline ground state. We fully agree that bulk amorphous amoxicillin represents a high-energy metastable form, while the crystalline form is the thermodynamic minimum in an unconstrained environment.

However, nanocrystal formation inside our FCC particles is governed by local energy minima defined by nanoscale confinement, not by the unconstrained bulk thermodynamic landscape. Several mechanisms can in principle drive nanocrystal formation, including (i) rapid concentration changes, (ii) rapid solvent removal, (iii) surface crowding during sorption, (iv) self-assembly driven by intermolecular interactions, and (v) spatial confinement within nanoscale pores. While multiple effects may contribute, we believe mechanism (v) is the dominant one in our system.

The intraparticle pore network in the FCC materials contains a large fraction of pores <100 nm, meaning that the system can only reach a pore-size-limited local minimum, in which nanocrystals are the only crystallite size physically accommodated. This nanoscale confinement prevents growth toward the fully developed bulk crystal lattice, thereby stabilizing small crystallites or partially ordered domains even though a larger crystal would be the global low-energy state under bulk conditions. The absence of long-range ordering or superlattice reflections in the nano-XRD data further supports spatial confinement rather than free-surface self-assembly as the governing mechanism.

Relevant examples of spatially confined nanocrystal formation under nanoscale geometric restriction have been reported in the literature (e.g., Lee et al., Acc. Chem. Res. 2018, 51, 2867–2879; <https://pubs.acs.org/doi/10.1021/acs.accounts.8b00338>), and surface-driven self-assembly yielding ordered superlattices typically produces distinct superlattice periodicities in diffraction patterns (e.g., Liu et al., Nanoscale Research Letters 2021, 16, 172). Such features were not observed in our nano-XRD measurements, consistent with nanoscale confinement rather than 2D surface ordering.

We have now revised the text to clarify this point and avoid any ambiguity around the use of “lowest energy” in the context of a confined system.

2) We thank the reviewer for this insightful question. We did not perform direct structural analysis of the amoxicillin during or after the long-term release studies, as the film samples were not preserved under conditions that would allow reliable post hoc characterization. However, the release behaviour strongly suggests that crystallization during dissolution is unlikely to be the dominant factor. The initial burst phase corresponds to dissolution of the surface-accessible nanocrystalline fraction already present before release, while the subsequent months-long slow release aligns with the confined amorphous deposits identified by SEM and XRD. Because the amorphous fraction is embedded within nanoscale pores, any transformation into larger crystalline domains would be sterically restricted. Thus, while we cannot exclude minor recrystallization during release, the observed profile is most consistently explained by the coexistence of pre-existing surface nanocrystals and intrapore amorphous amoxicillin rather than by crystal growth occurring during dissolution. We have now added a short clarifying statement in the manuscript following the discussion of Figs. 8–9 to make this explicit.

3) It is well known that amorphous drug solids have higher apparent solubility and faster dissolution rates than their crystalline counterparts because the absence of a crystal lattice lowers the energetic barrier for solvation. In our FCC-loaded system, the intrapore amorphous amoxicillin fraction is therefore expected to dissolve more readily when the release medium penetrates the pores, whereas surface-deposited nanocrystals dissolve more slowly. Thus, the presence of both forms likely underlies the observed biphasic release. If crystallization had occurred during formulation or storage, this would likely have diminished the solubility advantage and slowed release; the structural analyses (SEM and XRD) confirm that a significant amorphous fraction was indeed present, supporting the enhanced release behaviour observed. We have now added a short clarifying statement in the manuscript.
