# Supplementary material for: Functionalized calcium carbonate microparticles in ethyl cellulose films: A vehicle for sustained amoxicillin release for medical applications
Source: PLoS One. 2026 Apr 2;21(4):e0320280. doi: 10.1371/journal.pone.0320280 (PMC13046161; doi:10.1371/journal.pone.0320280)
Supplement: Attachment — Submitted filename: Response_to_reviewers_auresp_4.docx [file pone.0320280.s016.docx]

**6. Review Comments to the Author**

Reviewer #4: The authors have carefully and satisfactorily addressed the major points raised in the previous review. The revised manuscript is substantially clearer, particularly regarding the distinction and rationale for the different FCC materials, the positioning of TGA versus UV–vis measurements for drug release, and the discussion of novelty relative to existing amoxicillin delivery systems. Terminology is now consistent, and the scope of the work is appropriately framed as a proof-of-concept study focused on sustained release behavior.

While mechanical and swelling properties of the films and statistical replication of long-term release experiments remain outside the scope of the present work, these limitations are now explicitly acknowledged and no longer overstated in the conclusions. The experimental data presented support the claims made within this defined scope.

Only minor points remain, mainly editorial in nature (e.g., wording, figure labeling, and formatting).

Detailed comments and suggestions are provided in the attached review letter. These points do not affect the scientific validity of the study.

Overall, the manuscript is technically sound and suitable for publication after minor editorial adjustments.

**Remaining Issues and Minor Comments**

**1. Statistical and quantitative language**

Long-term release experiments are still based on single representative samples. While

this limitation is acknowledged, language such as “average release rate” should be

softened to avoid over-interpretation. Terms such as “apparent” or “estimated” release

rate would be more appropriate in this context.

**2. Figure clarity**

Figures 8 and 9 would benefit from clearer, more explicit labeling of “loaded” versus

“physical mixture” directly on the plots to improve readability without requiring

extensive reference to the captions or text.

**3. Formatting and presentation**

A small number of formatting artifacts and spacing inconsistencies remain in the revised

manuscript and should be corrected during final editing.

**4. MIC and biological relevance**

The discussion linking sustained release behavior to MIC values is informative; however,

it should remain cautiously framed as indicative only. Care should be taken to avoid

implying predictive relevance for in vivo efficacy at this stage.

These points do not affect the scientific validity of the study.

Overall, the manuscript is technically sound and suitable for publication after minor editorial adjustments.

**Response to reviewer #4**

We thank the reviewer for these constructive and helpful final comments, which have improved the clarity of the manuscript.

**Remaining Issues and Minor Comments**

**1. Statistical and quantitative language**

We agree and have revised the manuscript to replace “average release rate” with “estimated release rate” to reflect that the long-term release data are based on single representative samples**.**

**2. Figure clarity**

We have retained the legend and added explicit curve labels directly within Figures 8 and 9 to clearly distinguish “loaded” and “physical mixture” samples.

**3. Formatting and presentation**

We have carefully reviewed the manuscript and corrected remaining formatting artifacts and spacing inconsistencies during final editing.

**4. MIC and biological relevance**

We agree and have revised the MIC discussion to ensure it is framed as indicative only and does not imply predictive in vivo efficacy.

**Reviewer #6:** All comments have been addressed satisfactorily. No further questions. I can now recommend publishing.

**Response to Reviewer #6**

We thank the reviewer for their careful evaluation and for confirming that all comments have been satisfactorily addressed.
